# Astrocyte Ca^2+^ Dysregulation in Alzheimer’s Disease Mouse Models: Revisiting the Dogma of Hyperactivity

**DOI:** 10.3390/biom16030404

**Published:** 2026-03-10

**Authors:** Alessandro Di Spiezio, Micaela Zonta

**Affiliations:** 1 Neuroscience Institute, National Research Council (CNR), 35131 Padua, Italy; alessandrodispiezio@cnr.it; 2 Padova Neuroscience Center, University of Padua, 35129 Padua, Italy

**Keywords:** astrocytes, Alzheimer’s disease, Ca^2+^ signaling, Alzheimer’s disease animal models, amyloid β

## Abstract

Astrocytes are essential gatekeepers of brain homeostasis, and the disruption of their functions can contribute to the development of several neurological diseases. Among astrocyte signaling pathways, the intracellular second messenger Ca^2+^ plays a pivotal role in regulating the release of gliotransmitters, which actively modulate fundamental processes in the brain such as synaptic plasticity and memory function. Several studies over the years support the idea that dysregulated astrocytic Ca^2+^ homeostasis represents a relevant mechanism in Alzheimer’s disease pathogenesis. Early works in transgenic mice modelling Alzheimer’s disease reported increased Ca^2+^ activity in astroglial cells, supporting the idea of hyperactivity as a common trait of astrocytes in this pathology. However, recent studies have described astrocyte Ca^2+^ hypoactivity in various mouse models, revealing a more complex and heterogeneous scenario. In this review, we summarize and critically discuss the main studies addressing the direction(s) of astrocytic Ca^2+^ signaling dysfunction in mouse models of Alzheimer’s disease. We prioritize investigations performed in ex vivo and in vivo conditions, carefully comparing the different experimental approaches used to measure Ca^2+^ activity in astrocytes. By integrating results across multiple mouse models and methodological strategies, we aim to provide a more complete picture of astrocyte Ca^2+^ dysregulation in Alzheimer’s disease.

## 1. Astrocytes and Ca^2+^ Signaling Pathways

Astrocytes have been initially acknowledged to play a merely trophic and supportive role in the central nervous system. However, during the past three decades, accumulating evidence has demonstrated that these cells are central regulators of brain homeostasis and actively participate in key physiological processes, including the modulation of synaptic transmission, neurovascular coupling, ion buffering and immune responses [1,2,3,4,5].

Astrocytes express a plethora of receptors that allow them to sense changes in their surrounding environment. As for the other cell types, the activation of these receptors triggers various intracellular signaling pathways, depending on the specific receptor involved and its physiological function [6,7]. Among the various intracellular pathways, Ca^2+^ signaling in astrocytes has emerged as a crucial mediator of many of their functions, particularly in regulating gliotransmitter release and neuron–glia communication. Indeed, increases in astrocytic Ca^2+^ concentration can trigger the release of a variety of gliotransmitters, including glutamate, GABA, ATP, D-serine and BDNF, which modulate synaptic transmission and neuronal excitability [8,9,10,11,12,13,14,15]. Through Ca^2+^-dependent activity, astrocytes influence both pre- and postsynaptic terminals, regulating neurotransmitter release probability, receptor activation and synaptic strength, ultimately shaping memory processes [6]. Consequently, astrocytic Ca^2+^ signaling emerges as a fundamental mechanism linking cellular activity to circuit level up to behavioral outcomes. Thanks to the development of chemical and genetically encoded Ca^2+^ indicators, studying astrocytic Ca^2+^ signaling has historically become a standard method to assess astrocyte activity or functionality [16,17]. Astrocytes rely on several mechanisms to activate intracellular Ca^2+^ signaling (for a recent review, see [18]). One major pathway involves G protein-coupled receptors (GPCRs) [6], also known as metabotropic receptors. GPCRs can be coupled to three subtypes of G protein (Gq, Gs and Gi), differently linked to the intracellular second messengers Ca^2+^ and cyclic adenosine monophosphate (cAMP) [19].

In the present review, we will focus on Gq receptors, mentioning metabotropic glutamate receptors (mGluRs), purinergic receptors (P2YRs) and adrenergic receptors (ARs). Upon ligand binding, Gq receptors activate phospholipase C (PLC), which, in turn, converts phosphatidylinositol 4,5-bisphosphate (PIP_2_) into inositol 1,4,5-trisphosphate (IP_3_) and diacylglycerol (DAG). IP_3_ then binds to IP_3_ receptors (IP_3_Rs) on the endoplasmic reticulum (ER), promoting channel opening and the release of stored Ca^2+^ into the cytosol. This massive release of Ca^2+^ from the ER triggers a mechanism known as store-operated Ca^2+^ entry (SOCE): stromal interaction molecules (STIMs) sense ER depletion and recruit plasma membrane Ca^2+^ release-activated Ca^2+^ (CRAC) channels, formed by ORAI proteins, allowing Ca^2+^ influx from the extracellular space. In addition to metabotropic signaling, astrocytes also express ionotropic receptors and various ion channels that permit direct ion flux across the plasma membrane. Some of these, such as TRPA1 and the mechanoreceptor Piezo1, are Ca^2+^ permeable receptors mediating extracellular Ca^2+^ influx upon activation. Other mechanisms leading to intracellular Ca^2+^ increases involve ion exchangers such as the Na^+^/Ca^2+^ exchanger (NCX). Activation of the glutamate transporter 1 (GLT-1) leads to Na^+^ influx into the cytoplasm, which forces NCX into the reverse mode, promoting Ca^2+^ entry from the extracellular space. On this note, such Ca^2+^ influx, as well as Ca^2+^ release from IP_3_Rs, can trigger a process known as Ca^2+^-induced Ca^2+^ release (CICR), in which the rise of the cytosolic Ca^2+^ concentration activates calcium-binding sites in ryanodine receptors (RyRs) and in activated IP_3_Rs on the ER, resulting in regenerative Ca^2+^ release from internal stores [20,21].

## 2. Alzheimer’s Disease: Overview and Mouse Models

Astrocytic dysfunction has been increasingly implicated in the pathogenesis of numerous neurological and neurodegenerative disorders, including Alzheimer’s disease (AD). AD is a progressive neurodegenerative condition characterized by a cognitive decline and memory issues, representing the most common form of dementia, currently affecting over 40 million individuals worldwide as reported by the Alzheimer’s Disease International (www.alzint.org accessed on 1 March 2026). Histologically, AD is characterized by the extracellular accumulation of amyloid beta (Aβ) plaques and the intracellular formation of neurofibrillary tangles composed by hyperphosphorylated tau protein. These pathological hallmarks are associated with synaptic dysfunction, neuronal loss and neuroinflammation [22]. Despite decades of intensive research, the precise pathogenic mechanisms underlying AD remain poorly understood, significantly hindering the development of effective disease-modifying therapies.

It is estimated that only 1–5% of AD patients carry genetic mutations directly associated with early-onset familial AD (EOFAD). These mutations affect three genes, coding for the amyloid precursor protein (APP) and for presenilin 1 and 2 (PS1 and PS2). Presenilins are part of the catalytic unit of γ-secretase, the enzyme responsible for the C-terminal cleavage of Aβ peptides. Mutations in one or more of these genes in AD patients drive an increase in the production of insoluble Aβ species [23,24,25,26]. The majority of AD cases, conversely, do not present distinctive genetic mutations and are therefore classified as sporadic, indicating a multifactorial etiology that includes environmental, metabolic and lifestyle-related risk factors and underlies late-onset AD (LOAD). Among sporadic AD, the presence of the apolipoprotein E variant ApoE4 represents the major genetic risk factor increasing the probability to develop the pathology [27]. This complexity further hampers the identification of a unified pathogenic pathway suitable as therapeutic target.

**Figure 1:**
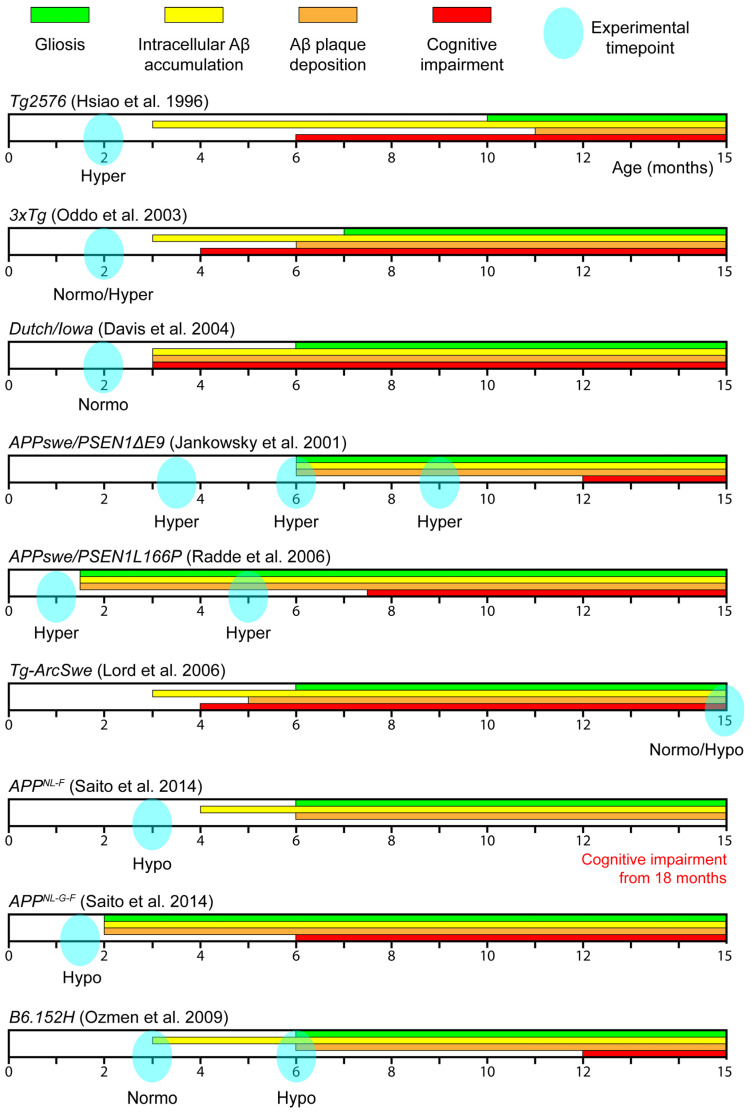
Schematic representation of the phenotypes displayed by the Alzheimer’s disease mouse models described in the present review [28,29,30,31,32,33,34,35]. For each mouse line, the onset of gliosis, intracellular neuronal Aβ accumulation, Aβ plaque deposition and cognitive impairment is indicated by a color-coded bar starting in correspondence of the onset age. Arrows mark the age analyzed in the studies and specify the direction of astrocytic Ca^2+^ dysfunction.

To model the disease and explore its mechanisms, various transgenic mouse lines harboring EOFAD mutations have been developed. Although these animal models fail to fully recapitulate the pathological features of the human disease, they can provide valuable insights into the cellular and molecular mechanisms underlying AD. To date, relatively few studies have investigated astrocytic Ca^2+^ activity in the context of AD. In this review, we summarize all available studies that address this topic using ex vivo or in vivo approaches. Specifically, we find 10 studies examining astrocytic Ca^2+^ activity in nine different mouse lines. The information on these mouse lines is also available on Alzforum (www.alzforum.org/research-models accessed on 1 March 2026) and graphically summarized in Figure 1.

- *Tg2576*

*Tg2576* mice overexpress in heterozygosis a mutant form of human APP (isoform 695) with the Swedish mutation (KM670/671NL), which, per se, increases total Aβ production, under the control of the hamster prion protein (PrP) promoter. The high levels of APPswe (5-fold above the physiological level) lead to elevated levels of Aβ_1–40_ and Aβ_1–42_ between 3 and 9 months of age and amyloid plaque deposits at 11 months of age. Regarding cognitive decline, the results are divergent and report memory impairment already at 6 months or starting from 12 months of age. Microglia activation is observed at 10 months of age [28,36].

- *3xTg*

*3xTg* mice, firstly described in 2003, present the following three mutations in homozygosis: the Swedish K670N/M671L mutation in the human APP gene, the P310L mutation in the Microtubule-Associated Protein Tau (MAPT) gene, both under the control of the mouse Thy1.2 promoter, and the M146V mutation in the PSEN1 gene, under the PSEN1 endogenous promoter. Although MAPT mutation is not associated with EOFAD, it was introduced in this model to produce tangle pathology in transgenic animals. Intracellular Aβ accumulation starts at 3–4 months of age, together with cognitive impairment, while extracellular Aβ deposits appear by 6 months in the frontal cortex. Reactive astrogliosis and microglia activation are observed at 7 months of age. Tau pathology occurs later, with aggregates of conformationally altered and hyperphosphorylated tau detected in the hippocampus of 12–15 months old transgenic mice [29,37].

- *Tg-SwDI (Dutch/Iowa)*

*Tg-SwDI* mice harbor three mutations in heterozygosis on the human APP gene: the Swedish K670N/M671L (APPswe), the Dutch E693Q and the Iowa D694N mutations, all under the control of the mouse Thy1.2 promoter. These transgenic animals show plaque-like structures associated with increased levels of soluble forms of Aβ species as early as month 3, with fibrillary amyloid deposits in the cerebral microvasculature starting at 6 months of age. Cognitive decline is observed starting from 3 months and gliosis at 6 months of age [30,38].

- *APPswe/PSEN1ΔE9 (APP/PS1)*

*APPswe/PS1ΔE9* mice were created by co-injecting two different vectors expressing human APPswe and PSEN1 lacking exon 9 under the control of mouse prion protein promoter. In hemizygous mice, plaques deposition and reactive astrogliosis start at 6 months of age and worsen with age. Cognitive impairment is significant at 12 months of age, although some groups reported small differences already at 6 months of age [31,39,40,41].

- *APPswe/PSEN1L166P (APPPS1-21)*

*APPswe/PSEN1L166P* mice express human APPswe and PSNE1 carrying the L166P mutation, both under the Thy1 promoter. Hemizygous *APPswe/PSEN1L166P* mice are characterized by overexpression of human APP (3-fold above the endogenous murine gene) and are highly amyloidogenic (due to a high Aβ_42_/Aβ_40_ ratio), with plaque deposition and gliosis starting already at 6 weeks. Despite the high levels of Aβ_42_ and amyloid plaques, cognitive impairment manifests only between 7 and 8 months of age [32,42,43].

- *Tg-ArcSwe*

*Tg-ArcSwe* mice harbor human APP carrying the Swedish (KM670/671NL) mutation and the Arctic mutation (E693G) in heterozygosis under the control of the murine Thy1 promoter. The arctic mutation aggravates the amyloid pathology of the *Tg2576* mice, leading to earlier plaque deposition (at 5–6 months of age) and to cognitive decline starting at 4 months of age. This model is characterized by very early (month 1) accumulation of intraneuronal Aβ, elevated CSF Aβ levels and detectable CSF tau protofibrils at 4 months of age. Plaque composition and morphology are different compared to the *Tg2576* line, with plaques displaying more homogeneity in terms of structure and size [33,44,45].

- *APP^NL-F^*

All mouse models based on APP mutations described so far rely on overexpression of human APP accompanying the endogenous murine protein. To overcome the possible artefact deriving from APP overexpression, Saito and colleagues created two different mouse lines expressing the humanized mutated APP gene under the control of the endogenous mouse APP promoter, thus conferring appropriate cell type and temporal specificity to APP expression. Homozygous *APP^NL-F^* mice carry the Swedish “NL” and the Iberian “F” (I716F) mutations, which influence the activity of secretases, resulting in an increase in total Aβ production and Aβ_42_/Aβ_40_ ratio, respectively. Amyloid plaque deposition and gliosis start from 6 months of age in both the cortex and hippocampus, and notably, hyperphosphorylated tau is found in dystrophic neurites around plaques. Cognitive impairment appears at around 18 months of age, though in the absence of neurodegeneration [34].

- *APP^NL-G-F^*

The second mouse line developed was *APP^NL-G-F^*. In addition to the Swedish and Iberian mutations, *APP^NL-G-F^* mice carry the Arctic mutation, which makes the pathology more severe by promoting Aβ aggregation through enhanced oligomerization and reduced proteolytic degradation. Amyloid plaque deposition begins as early as month 2, accompanied by gliosis, while cognitive deficits typically appear around 6 months of age [34].

- *B6.152H (PS2APP)*

Two different transgenic mouse lines carry the APPswe mutation under the control of the Thy1.2 promoter and the Volga (N141I) mutation in PSEN2 driven by the mouse prion protein promoter. These lines differ in their method of generation, resulting in different levels of APP expression. One *PS2APP* model has been obtained by crossing *PS2* (N141I) mice with *APPswe* mice [46], thus expressing the mutations in heterozygosis, while the *PS2APP* mouse line known as *B6.152H*, described in this review, has been developed by co-injecting the two mutated constructs into *C57BL/6* zygotes and is maintained as a homozygote line, resulting in higher expression of transcripts [35]. *B6.152H* mice display less variability in pathology expression and develop amyloid plaques at 6 months of age, along with gliosis, while cognitive deficits appear at 8 months of age [35,47].

## 3. Astrocytic Ca^2+^ Dysregulation in AD

Nowadays, many studies support the idea that dysregulated astrocytic Ca^2+^ homeostasis is a relevant feature of AD pathogenesis. However, it is still unclear and under debate whether astrocytes exhibit increased or decreased Ca^2+^ activity in AD, whether this dysregulation is an early event preceding plaque deposition or a direct consequence of plaque accumulation and what the molecular mechanisms underlying Ca^2+^ homeostasis dysfunction are. Literature reviews on this topic largely indicate a clear trend toward increased Ca^2+^ activity, mirroring the neuronal hyperexcitability that characterizes the early stages of pathology. This predominant view mainly stems from early reports on *Tg2576* and *APP/PS1* transgenic mice (see above for details on strains), which revealed a higher level of spontaneous Ca^2+^ activity and the occurrence of intercellular cortical Ca^2+^ waves linked to over-activation of purinergic receptors. Recent studies, though, provided novel information that widens our perspective on the “direction” of signal disruption in astrocytes from AD models.

In this review, we summarize the studies that addressed these questions, focusing primarily on those employing ex vivo and in vivo imaging techniques, aiming to identify a consensus among the different protocols, age and AD mouse models used (Table 1). Astrocyte phenotype has been classified as “hyperactivity”, “normoactivity” or “hypoactivity”, indicating higher, equivalent or lower spontaneous Ca^2+^ signals in AD mice compared with control mice. “Hyporesponsiveness”, on the other hand, denotes lower stimulus-evoked Ca^2+^ signals.

### 3.1. Astrocytic Ca^2+^ Dysregulation in AD: Hyperactivity

Takano and colleagues from the Nedergaard lab performed seminal work on astrocytic Ca^2+^ dysregulation in AD in 2007 while investigating brain vasculature involvement in the early stages of AD in living animals [48]. The crucial role of astrocytes in controlling vasodilation and vasoconstriction had been discovered a few years earlier; thus, the group also analyzed astrocyte Ca^2+^ signals in three different AD mouse models. The study focused on *Tg2576*, *3xTg* and *Tg-SwDI* mice at 2–4 months of age, before the onset of extracellular amyloid deposition, neuronal degeneration and gliosis. Chemical Ca^2+^ indicator Fluo-4 AM and two-photon (2P) microscopy were used to image cell bodies and major processes in astrocytes from cortical layer II in ketamine/xylazine-anesthetized animals.

The authors observed a striking elevation in the frequency of spontaneous intracellular Ca^2+^ activity in *Tg2576* mice relative to wild-type (*WT*) controls (0.4 events/min in *WT* vs. 2.3 events/min in *Tg2576*). However, this finding was not consistently observed in the other transgenic mouse models examined. Specifically, *3xTg* mice showed mixed effect: while most mice did not differ from *WT* controls, a few animals displayed increased astrocyte Ca^2+^ activity (0.4 events/min in *WT* vs. 0.9 events/min in “*3xTg* low activity” vs. 3.5 events/min in “*3xTg* high activity”). The third mouse line (*Tg-SwDI*) showed no difference in Ca^2+^ oscillations compared to littermate *WT* controls (0.4 events/min in *WT* vs. 0.7 events/min in *Tg-SwDI*). Interestingly, when these mice were injected intravenously (i.v.) with Aβ_40_ peptide, astrocytes responded with a significantly greater increase in oscillation frequency compared to glial cells in their littermate controls. Collectively, this was the first in vivo evidence that astrocytic Ca^2+^ activity can be dysregulated in AD, although with different phenotypes depending on the mouse model.

Dysregulation of astrocyte Ca^2+^ signals in *Tg2576* mice was investigated also in a study by Pirttimaki and colleagues, from the Parri lab, reporting a strong increase in astrocyte spontaneous activity (0.09 events/min in *WT* vs. 0.36 events/min in *Tg2576*) also in hippocampal slices from younger animals (PN4-14) [49].

In 2009, Kuchibhotla and colleagues from the Backsai lab conducted an in-depth characterization of Ca^2+^ homeostasis in cortical astrocytes from *APPswe/PS1ΔE9* transgenic mice [50]. To focus on the chronic phase of amyloid pathology, the authors performed their study on isoflurane-anesthetized animals at 6–8 months of age, a time point corresponding to significant Aβ plaque burden in this model. They employed the Ca^2+^ indicator Oregon Green BAPTA-1 (OGB-1) to combine fluorescence lifetime imaging microscopy (FLIM) with classical 2-photon Ca^2+^ imaging, allowing for quantification of both the resting intracellular Ca^2+^ concentration and spontaneous Ca^2+^ oscillations in astrocyte somata.

The authors reported significantly elevated basal intracellular Ca^2+^ concentrations in astrocytes from *APPswe/PS1ΔE9* mice compared to *WT* controls (81 nM in *WT* vs. 149 nM in *APPswe/PS1ΔE9*). AD mice were also characterized by an increase in both the percentage of astrocytes displaying spontaneous Ca^2+^ transients (8.1% in *WT* vs. 27.9% in *APPswe/PS1ΔE9)* and the amplitude of these signals (23.2 ΔF/F in *WT* vs. 33.6 ΔF/F in *APPswe/PS1ΔE9*). The increase in the fraction of active astrocytes was unaffected by the blockade of neuronal firing through TTX application. Notably, spontaneous hyperactivity was absent at an earlier disease stage (3–3.5-month-old AD mice) lacking amyloid plaque deposition, suggesting that Ca^2+^ dysregulation correlates with disease progression. In addition, the authors observed the occurrence of peculiar intercellular Ca^2+^ waves that originated from astrocytes located near amyloid plaques (within 25 μm) and propagated radially for distances up to 200 μm. However, higher amplitude Ca^2+^ signals were observed in individual astrocytes independently of plaque proximity. Based on these findings, the authors proposed a direct modulatory effect of Aβ plaques on global astrocytic function.

The presence of pathological Ca^2+^ waves was also reported in 2014 in *APPswe/PSEN1L166P* mice, an AD model characterized by faster progression of amyloid pathology, with plaque deposition occurring at 6 weeks of age. Delekate and colleagues, from the Petzold lab, investigated astrocytic Ca^2+^ activity in the somatosensory cortex of 5–9-month-old isoflurane/ketamine-anesthetized animals, by employing the Ca^2+^ indicator OGB-1 and the intravital marker methoxy-XO4 to image amyloid plaques [51]. Consistent with previous findings, this study reported that transgenic astrocytes exhibit elevated somatic Ca^2+^ activity. The frequency of somatic Ca^2+^ transients was used to classify astrocytes as inactive (0 events/min), active (0.1–0.4/min) or hyperactive (>0.4/min), revealing that 33.8% of AD astrocytes displayed hyperactivity compared to 4.1% in the littermate controls. Notably, the study investigated spontaneous activity in dye-loaded astrocytic endfeet, showing that hyperactivity similarly involves these perivascular processes. Also in this model, the increase in astrocyte activity was independent of neuronal firing. However, the authors demonstrated that proximity to amyloid plaques (within 50 µm) significantly increased the fraction of hyperactive and active astrocytes. Notably, the previous study [50] analyzed the correlation of plaque proximity with the increase in signal amplitude but not in signal frequency in astrocytes, thus hampering the comparison of these results. It is noteworthy to underline that the term “hyperactivity” is manifestly an umbrella term that can cover different parameters of Ca^2+^ activity in astrocytes. The Petzold group further investigated the molecular pathways underlying the increased Ca^2+^ activity and the propagation of Ca^2+^ waves in space and time, focusing on the role of purinergic signaling and specifically on P2Y1 receptors, highly upregulated in astrocytes surrounding amyloid plaques. Topical application of a general P2Y receptor antagonist (PPADS, 500 µM) or a P2Y1-specific antagonist (MRS2179, 500 µM) was sufficient to restore Ca^2+^ activity to levels comparable to littermate control and to block Ca^2+^ wave propagation. Pharmacology experiments revealed the involvement of ER-dependent Ca^2+^ release and connexin hemichannels in astrocyte hyperactivity.

The same group, years later, published another study [52] demonstrating that chronic (6 weeks) administration of P2Y1R inhibitors (MRS2179 1 mM or BPTU 10 µM), applied through i.c.v. osmotic minipumps, normalized astrocytic activity and reduced neuronal hyperexcitability in the somatosensory cortex. Notably, the authors disclosed astrocyte hyperactivity also in the dorsal hippocampus, using the genetically encoded Ca^2+^ indicator GCaMP6f. Despite the use of a genetically encoded Ca^2+^ indicator, the analysis was limited to cell somata and missed checking the recovery of physiological astrocyte activity upon chronic P2Y1R inhibition. On the other hand, the study analyzed the defects of synaptic plasticity in hippocampal slices from *APP/PS1* mice and demonstrated that MRS2179 treatment can rescue impaired hippocampal LTP and prevent cognitive decline, suggesting it may be a promising therapeutic candidate.

In a study published by Bosson and colleagues from the Albrieux lab in 2017, a different brain region and an earlier developmental time point were investigated in the same AD mouse model (*APPswe/PSEN1L166P*) [53]. Specifically, the authors performed Ca^2+^ imaging of astrocytes in the CA1 stratum radiatum of hippocampal slices from young mice (PN19-28) and their WT littermates. Their analysis thus focused on the early stage of the disease, characterized by increased Aβ_1–42_ levels but preceding both the deposition of amyloid plaques and the appearance of reactive astrogliosis.

To investigate astrocytic Ca^2+^ activity, two imaging strategies were employed: (1) bulk loading of Fluo-4 AM across the entire hippocampal slice allowed visualization of a large population of astrocytes, primarily capturing large somatic Ca^2+^ events, and (2) whole-cell patch-clamp dye loading of individual astrocytes enabled resolution of principal processes and thinner arborizations, manually segmented in subregions that the authors term “microdomains”, although they represent only a minor fraction of the bushy ensemble of astrocyte microdomains. The key focus of the study was the TRPA1 channel, whose expression was found to double between P19 and P30 in *APPswe/PSEN1L166P* mice, in coincidence with the onset of Aβ secretion. Despite a similar proportion of active astrocytes between *APPswe/PS1L166P* and *WT* littermates, bulk-loaded astrocytes from the AD model displayed a significantly higher frequency of Ca^2+^ events (0.33 events/min in *WT* vs. 0.67 events/min in *APPswe/PS1L166P*), shifting the frequency distribution toward a hyperactive (>0.6 events/min) population (6% in *WT* vs. 21% in *APPswe/PS1L166P*). Notably, both event frequency and the fraction of hyperactive cells were fully reversed to *WT* values by pharmacological blockade of TRPA1 with HC030031 (40 μM). Subregion analysis in patch-loaded astrocytes revealed a low but statistically significant increase in event frequency in *APPswe/PS1L166P* mice. TRPA1 inhibition normalized the frequency increase (0.40 events/min in *WT* vs. 0.45 events/min in *APPswe/PS1L166P* vs. 0.43 events/min *APPswe/PS1L166P* + HC) and reduced the proportion of active microdomains in *APPswe/PS1L166P* (55.0% in *WT* vs. 65.7% in *APPswe/PS1L166P* vs. 29.3% *APPswe/PS1L166P* + HC).

Interestingly, bath application of Aβ oligomers (100 nM) to *WT* hippocampal slices increased both the number of active astrocytes and the percentage of the territory engaged in Ca^2+^ activity; this effect was abolished by HC030031. These experiments suggest that Ca^2+^ dysregulation may be an early event in AD pathogenesis and that soluble Aβ oligomers play a crucial role in modulating astrocyte Ca^2+^ activity and in the establishment of an astrocyte phenotype possibly concurring to neurotoxicity.

### 3.2. The Turning Point: Hyperactivity and Hyporesponsiveness

The study performed in 2021 by Lines and colleagues from the Araque and Kofuji lab [54] aimed to characterize astrocytic Ca^2+^ dynamics beyond the soma in the *APPswe/PS1ΔE9* mouse model, using GCaMP7 and differentiating somatic signals from events within “arborizations”. Although this genetically encoded Ca^2+^ indicator allows the visualization of processes and microdomains, it is noteworthy that the analysis was performed on astrocyte territories identified by thresholding sulforhodamine 101 (SR101) fluorescence, thus missing a conspicuous part of microdomains, similarly to what obtained in the work of Bosson and colleagues through individual loading of astrocytes with Fluo-4.

The authors crossed *APPswe/PS1ΔE9* mice with a mouse line expressing GCaMP7 under the specific astrocyte GLT1 promoter and analyzed 6–9-month-old mice, at a disease stage subsequent to plaque deposition, and control littermates. Complementing previous findings describing higher signal amplitude in astrocytes from the same AD model [50], this study reported an increased frequency of spontaneous Ca^2+^ signals in both somata and arborizations of astrocytes from the somatosensory cortex of urethane-anesthetized *APPswe/PS1ΔE9* mice. The authors also examined the relationship between Ca^2+^ activity and the presence of amyloid plaques, revealing that Ca^2+^ events in astrocytic processes, but not in somata, were more frequent in cortical regions with higher plaque density. However, the frequency of Ca^2+^ events did not correlate with the distance to the nearest plaque, suggesting that astrocytic hyperactivity is not directly influenced by plaque proximity. Rather, it may depend on the concentration of soluble Aβ, the stage of disease progression or other factors within the pathological milieu. In parallel, the authors monitored sensory-evoked astrocytic Ca^2+^ responses following electrical stimulation of the contralateral hind paw, performing the first assessment of evoked Ca^2+^ signals in astrocytes from an AD model. In AD mice, the responses exhibited aberrant dynamics characterized by delayed onset, prolonged rise time and slower decay at both somatic and arborization levels. Besides confirming spontaneous astrocyte hyperactivity, this study thus reveals that astrocyte recruitment by sensory stimulation is slower and dysregulated in *APPswe/PS1ΔE9* mice. Furthermore, sensory-evoked responses were shown to negatively correlate with local plaque burden and to be influenced by plaque proximity. Specifically, increasing the distance from plaques was associated with higher astrocyte recruitment by sensory stimulation, in terms of the fraction of activated astrocytes and percentage of astrocyte arborization involved in the response. Although a direct comparison of the amplitude of the response between *APPswe/PS1ΔE9* mice and *WT* littermates is not reported, related graphs suggest a lower amplitude of the response in astrocytes from AD mice. The authors conclude that the presence of Aβ plaques is associated with an increased spontaneous Ca^2+^ activity in astrocyte processes and with a lower response to sensory inputs, providing the first experimental evidence of hypofunction of astrocytes in AD mouse models.

### 3.3. Astrocytic Ca^2+^ Dysregulation in AD: Hypoactivity and Hyporesponsiveness

After many years of general agreement on astrocyte hyperactivity in AD mouse models, several publications have now confirmed that AD astrocytes can also exhibit Ca^2+^ hypoactivity.

In 2022, Åbjørsbråten and colleagues, from the Enger lab, combined 2P microscopy with virally delivered GCaMP6 to image astrocytes in the somatosensory cortex of awake, behaving 15-month-old *tg-ArcSwe* mice. This age corresponds to an advanced stage of disease, since plaque deposition, gliosis and cognitive impairment are present in 4–6-month-old animals in this AD model. The study investigated possible perturbations of the norepinephrine–astrocyte Ca^2+^ signaling axis [55]. Astrocytes respond with Ca^2+^ increases to noradrenaline in different brain regions via the activation of α1-ARs [56,57,58,59], and they have been specifically reported to display fast Ca^2+^ transients upon sensory and air-puff stimulation [60]. The authors used a sophisticated experimental paradigm in which mice were free to move on a treadmill while the Ca^2+^ signal and pupil dilation were simultaneously recorded. An air puff directed at the whiskers and nose was exploited to trigger a noradrenergic startle response, while pupil dilation was used as a readout of locomotion and startle. Event-based ROAs (regions of activity) were automatically detected in GCaMP-expressing astrocytes through an originally developed analysis suite and assigned to manually segmented astrocyte territories (soma, processes and endfeet) to discriminate the recruited territories. During quiet periods, the authors found no differences in astrocytic Ca^2+^ activity between *tg-ArcSwe* mice and *WT* littermate controls. Additionally, they observed rare, long-lasting pathological Ca^2+^ waves similar to those previously reported in *APP/PS1* mouse models, occurring in only 10–15% of the recordings. Spontaneous locomotor activity and startle response were accompanied by pupil dilation and increased astrocytic Ca^2+^ activity, computed as max ROA density and density rise time. During the startle response, the recruitment of Ca^2+^ activity in *WT* and *tg-ArcSwe* astrocytes was similar, but astrocyte Ca^2+^ response to locomotion was lower in *tg-ArcSwe* mice. In addition, pupil dilation and astrocyte responses were coupled in *WT* mice during both startle response and locomotion but uncoupled in AD mice. Notably, the authors found a significant reduction in noradrenaline release in the cortex of AD mice, thus suggesting that a defect in the degree of stimulation contributes the reduced response of astrocytes. Whether a diminished noradrenergic input is the main driver of the lower astrocyte response remains unclear, since the ability of astrocytes to respond to noradrenaline was not directly assessed in these mice.

Conversely, in the same year, Shah and colleagues from the De Strooper lab reported cell-autonomous dysregulation in astrocyte Ca^2+^ signals in an AD mouse model, investigating in a more translational study the relationship between resting-state functional connectivity (FC, measured via resting-state fMRI) and astrocytic Ca^2+^ signaling in the *APP^NL-F^* mouse model [61]. Firstly, the study monitored and compared FC and amyloid load in a cohort of cognitively healthy individuals scanned at two time points, revealing that an early increase in anterior–posterior cingulate FC characterized patients developing amyloid accumulation several years after, suggesting its potential as a very early functional biomarker. Driven by these results, the authors shifted the research to the *APP^NL-F^* mouse line, focusing on an early time point (3 months) preceding plaque deposition by a few months. Resting-state fMRI and 2P GCaMP6f imaging of Ca^2+^ dynamics in the cingulate cortex (CCx) of anesthetized animals confirmed the early increase in cingulate FC seen in patients and revealed a significant reduction in astrocytic activity in *APP^NL-F^* mice compared to *APP^NL^* controls (carrying only the APPswe mutation under its endogenous promoter). Specifically, there was a marked decrease in the percentage of active astrocytes (64% in controls vs. 28% in *APP^NL-F^*), in the frequency of Ca^2+^ events (0.12 peaks/min in controls vs. 0.04 peaks/min in *APP^NL-F^*) and in event amplitude (1.29 ΔF/F in controls vs. 1.21 ΔF/F in *APP^NL-F^*) in regions of interests (ROIs) encompassing both astrocyte soma and proximal processes. The authors report that Ca^2+^ activity in distal processes remains unaltered, but it is noteworthy that a single ROI comprising all distal territories was used to monitor distal Ca^2+^ signals of individual astrocytes, thus hampering an accurate assessment of the activity of discrete microdomains. Decreased astrocyte calcium signaling was observed at early stages of amyloid pathology also in a related, more severe AD mouse model, i.e., *APP^NL-G-F^* mice. The impairment of Ca^2+^ homeostasis in *APP^NL-F^* mice was associated with a marked reduction in IP_3_R2 in astrocytes, a finding consistent in both the *APP^NL-F^* mouse model and human AD brain tissue. Given the central role of astrocytes in regulating neuronal activity, the authors attempted to modulate intracellular Ca^2+^ signaling in *APP^NL-F^* mice by inducing AAV-mediated expression of Gq DREADDs (Designer Receptors Exclusively Activated by Designer Drugs) in astrocytes. Consistent with the observed reduction in IP_3_R2, the administration of the designer agonist CNO to activate DREADDs induced a weaker increase in Ca^2+^ signals in *APP^NL-F^* compared to control *APP^NL^* mice. Nonetheless, the increase in astrocytic Ca^2+^ activity triggered by CNO in *APP^NL-F^* mice was sufficient to normalize astrocytic Ca^2+^ signals and dampen neuronal hyperactivity. Of note, this mouse model also displays increased susceptibility to seizure and behavior hyperactivity during the light–dark cycle, and CNO application completely reversed these clinically relevant behaviors. Interestingly, DREADD activation in control *APP^NL^* mice induced astrocyte hyperactivity and promoted neuronal hyperactivity, seizure susceptibility and behavioral hyperactivity, demonstrating the importance of a tightly regulated Ca^2+^ activity in astrocytes.

In 2023, Lia and colleagues from the Carmignoto lab published a study reporting astrocyte hypoactivity in the *B6.152H* mouse model of AD [47]. The experiments were mainly performed in ex vivo brain slices from the somatosensory cortex of *B6.152H* mice, analyzing both spontaneous and evoked activity in astrocytes. Spontaneous signal was monitored also in vivo in isoflurane-anesthetized mice. Importantly, astrocyte-specific GCaMP6 expression and 2P microscopy allowed a detailed analysis of Ca^2+^ signals from all astrocyte territories, from the soma to principal processes to thin microdomains. The study investigated astrocyte activity at two stages of disease progression, before (3 months of age) and after (6 months of age) the onset of gliosis and plaque deposition in brain cortex. At the early stage, SSCx astrocytes exhibited a non-significant trend toward increased spontaneous Ca^2+^ activity at microdomains. However, this trend was reversed at 6 months of age, revealing a pronounced Ca^2+^ hypoactivity characterized by reduced active microdomains, frequency of events and event amplitude. This reduction in spontaneous activity was confirmed in vivo through 2P imaging of astrocytes from the somatosensory cortex of anesthetized *B6.152H* mice. Interestingly, spontaneous Ca^2+^ hypoactivity after plaque deposition was not influenced by the distance to plaques.

In analogy with the study of Lines, in which astrocyte response to sensory stimulation was probed, this work characterized, besides spontaneous activity, Ca^2+^ response to metabotropic agonists. In AD mice, after plaque deposition, astrocytes displayed hyporesponsiveness to ATP and noradrenaline (NA) at the level of all their territories, and this hypofunction was associated with both impaired NA-dependent, astrocyte-mediated LTP in the SSCx and defective tactile memory retention. The authors disclosed as a putative underlying molecular mechanism a significant reduction in ER Ca^2+^ content and astrocyte expression of STIM1, a key regulator of store-operated Ca^2+^ entry aimed at refilling ER. Interestingly, lower levels of STIM1 have been reported also in post-mortem tissue from sporadic AD patients [62]. Notably, astrocyte-specific overexpression of STIM1 via a lentiviral vector was successful in restoring both spontaneous and evoked Ca^2+^ activity in brain slices, as well in normalizing the impaired LTP observed in *B6.152H* mice. These results confirm how a dysregulation of astrocyte Ca^2+^ signals can affect brain physiology by altering synaptic plasticity.

In the same year, the work of Lee and colleagues from the Kastanenka lab analyzed the slow-wave activity (SWA) disturbances occurring one month before plaque appearance in young *APPswe/PS1ΔE9* mice, resembling those commonly reported in patients in early phases of AD [63]. The study disclosed a correlation between slow-wave disruption, sleep disturbances and a reduction of power in astrocyte Ca^2+^ transients occurring at the slow-wave frequency (0.2–1 Hz) in the SSCx of isoflurane-anesthetized AD mice. The reduction in amplitude was present in all astrocyte territories, but it was particularly significant at the level of microdomains. Optogenetic stimulation of ChR2-expressing astrocytes at the slow-wave frequency (0.6 Hz) was sufficient to restore SWA in transgenic mice, along with improving memory function. Although this study, unfortunately, did not investigate the degree of recovery of astrocyte Ca^2+^ signals upon optogenetic activation, the obtained results underscore the tight relationship between astrocyte hypoactivity and performance decline in brain function.

**Table 1:** Effects of AD-linked mutations on astrocytic Ca^2+^ activity in different mouse models of Alzheimer’s disease.

AD Model	Promoters	Age	Brain Region	Approach	Astrocyte Phenotype	Notes	Study
Tg-SwDI	Thy1.2 APPmut	2–4 months	SSX	In vivo anaesthetized (ketamine + xylazine)	Normoactivity	Hyperactivity when injected with Aβ_40_ i.v.	Takano et al. [48]
3xTg	Thy1.2-APPswe Thy1.2-MAPT endogenous PSEN1				Normoactivity Hyperactivity	Most astrocytes showed normoactivity, some others hyperactivity	
Tg2576	PrP-APPswe				Hyperactivity	Striking increase in event frequency	
		4–14 days	Hipp	Ex vivo hippocampal slices		Elevated Aβ_42_ levels in young Tg2576 mice; bath application of Aβ_42_ reduces hyperactivity	Pirttimaki et al. [49]
APPswe /PSEN1ΔE9	PrP-APPswe/PSEN1	6–8 months	Cortex	In vivo anaesthetized (0.5–1.0% isoflurane)	Hyperactivity	Normoactivity before plaques (3.5–4 months)	Kuchibhotla et al. [50]
		6–9 months	SSCx	In vivo anaesthetized (urethane 1.8 mg/kg)	Hyperactivity Hyporesponsiveness	Hyporesponsivness to n sensory stimulation	Lines et al. [54]
		4–6 months	SSCx	In vivo anaesthetized (1.75% isoflurane)	Hypoactivity	Reduced power in SWA-related Ca^2+^ oscillations	Lee et al. [63]
APPswe /PS1 L166P	Thy1-APPswe/PSEN1	5–9 months	SSCx	In vivo anaesthetized (0.4% isoflurane + ketamine)	Hyperactivity	Dependent on purinergic signal (P2YRs)	Delekate et al. [51,52]
		19–28 days	Hipp	Ex vivo hippocampal slices	Hyperactivity	Dependent on TRPA1 signaling	Bosson et al. [53]
tg-ArcSwe	Thy1-APPArcSwe	15 months	SSCx	In vivo awake	Normoactivity Hyporesponsiveness	Rare occurrence of waves; hyporesponsiveness linked to lower NA release	Åbjørsbråten et al. [55]
APP^NL-F^	endogenous APP	3 months	CCx	In vivo anaesthetized (medetomidine 0.05 mg/kg + 0.5% isoflurane)	Hypoactivity	Associated to IP_3_R2 downregulation	Shah et al. [61]
APP^NL-F-G^		6 weeks				Not further investigated	
B6.152H	Thy1-APPswe Prp-PSEN2	6 months	SSCx	Ex vivo cortical slices In vivo anaesthetized (isoflurane 0.5–0.8% + carprofen 5 mg/kg)	Hypoactivity Hyporesponsiveness	Associated to reduced ER Ca^2+^ content	Lia et al. [47]

## 4. Methodological Limitations

Despite technological advances and significant progress in the field, different methodological limitations can affect the experimental approaches reported in this review and used to monitor astrocyte Ca^2+^ dynamics. The first limitation concerns the use of chemical Ca^2+^ indicators, which primarily load cell bodies and principal processes of astrocytes. The majority of reports on astrocyte signals in AD models rely on chemical indicators and therefore do not provide information on the “bushy” arborization, which constitutes the larger fraction of astrocyte volume [64]. The development of GECIs allowed for spatially extending Ca^2+^ analysis by reliably capturing Ca^2+^ activity at the level of astrocytic microdomains [65].

Another major limitation of Ca^2+^ imaging studies is related to the use of anesthetics in the in vivo experiments. It is well known that anesthesia dampens both neuronal and astrocytic activity [66]. Therefore, although AD and control animals are anesthetized using the same protocol within each study, anesthesia-induced alterations in signaling may still represent a confounding factor worth considering. In this regard, lowering the levels of anesthetics may help reduce the differences in astrocyte Ca^2+^ signals between awake and anesthetized animals.

A further critical issue is that most studies assess astrocytic Ca^2+^ activity at only a single time point. This makes it difficult to determine whether hyper- or hypoactive phenotypes represent transient phases in disease progression or stable features of specific mouse models. This limitation could be overcome by performing Ca^2+^ imaging at multiple disease stages, ideally through longitudinal monitoring of Ca^2+^ activity in awake mice via chronic cranial windows or GRIN lenses coupled to miniaturized microscopes.

## 5. Discussion

A variety of mechanisms contributing to Ca^2+^ dysregulation in astrocytes emerges in the studies described in this review. Different works investigated the proximity of astrocytes to plaques as a possible factor contributing to cell impairment. Indeed, the presence of amyloid plaques as a common hallmark across AD models hints at insoluble amyloid aggregates as the major driving stimulus for astrocyte dysregulation. However, controversial results have been obtained on a possible correlation between plaque proximity and astrocyte Ca^2+^ signal impairment, in both hypo- and hyperactivity [47,50,51,54]. In addition, several studies have reported signal alterations in astrocytes also prior to the onset of plaque deposition [48,49,53,61,63], and a growing body of evidence now supports the hypothesis that soluble Aβ oligomers may be primarily responsible for some distinctive phenotypes observed in AD mouse models. Notably, already in 2012, Busche and colleagues demonstrated that soluble Aβ_42_ oligomers induce neuronal hyperactivity in the hippocampus of *WT* mice and are responsible of neuronal hyperactivity in *APPswe/PS1G384A* mice. Indeed, acute γ-secretase inhibition reduced soluble Aβ levels and restored physiological levels of activity in AD neurons [67]. Other studies suggest that not only neurons but also astrocytes exhibit altered Ca^2+^ signaling in response to Aβ exposure. For instance, Takano and colleagues revealed an increase in the frequency of Ca^2+^ oscillations in cortical astrocytes from *WT* and *Tg-SwDI* mice after intravenous administration of Aβ_1–40_ peptide (0.4 mg/kg). Pirttimaki and colleagues showed that both physiological (300 pM) and pathological (10 nM) concentrations of Aβ_1–42_ oligomers are sufficient to evoke intracellular Ca^2+^ transients in hippocampal astrocytes in ex vivo brain slices from *WT* mice. Furthermore, _Aβ_-induced astrocytic activity was completely abolished by the application of methyllycaconitine (MLA), an antagonist of the α7 nicotinic acetylcholine receptor subunit, highlighting the role of α7 receptors in mediating astrocytic responses to soluble Aβ oligomers [49]. Similar results were reported by Bosson and colleagues, who exposed hippocampal slices from *WT* mice to Aβ oligomers (100 nM). This treatment increased both the number of active astrocytes and the proportion of astrocytic territory engaged in Ca^2+^ activity, and this effect was abolished by the TRPA1 inhibitor HC030031 [53]. These data suggest that Ca^2+^ signaling can be affected by Aβ oligomers present in the early stages of Alzheimer’s disease and that multiple receptors may be involved, converging onto a common signaling pathway.

In addition to acute or chronic effects of Aβ_1–42_ oligomers on astrocytes, the occurrence of reactive astrogliosis in AD (and in other neurological disorders) has been suggested to influence astrocyte Ca^2+^ activity [68], for instance, through the upregulation of plasma membrane receptors. Reactive astrocytes re-express mGluR5 in pathophysiological conditions such as AD [69,70], epilepsy [71] and neuropathic pain [72]. Importantly, mGluR5 re-expression significantly enhances astrocytic Ca^2+^ activity, whereas its genetic silencing or pharmacological inhibition markedly reduces such activity in models of neuropathic pain and status epilepticus [72,73]. Other receptors involved in the inflammatory response, such as P2Y1 and TRPA1 receptors, are similarly upregulated in some AD mouse models, particularly in regions adjacent to amyloid plaques. Furthermore, pharmacological blockade of these receptors using specific antagonists has demonstrated beneficial effects, including improvements in behavioral outcomes [51,52,53,74].

Alongside alterations in the expression of plasma membrane receptors, animal models of AD can also display abnormalities in intracellular key modulators of Ca^2+^ signaling. Mutations in presenilins, which also localize in the ER membrane, can reduce the activity of the Sarco-Endoplasmic Reticulum Calcium ATPase (SERCA) that physiologically pumps Ca^2+^ ions from the cytosol to the ER [75,76]. In *B6.152H* mice, a diminished expression of STIM1, a key protein responsible for ER refilling following depletion, is accompanied by a reduction in ER Ca^2+^ content in astrocytes [47]. Interestingly, the expression of mutated PSEN2 alone was not sufficient to reduce Ca^2+^ signals in astrocytes [47], indicating that the concomitant presence of the APPswe mutation in *B6.152H* mice, which induces an earlier increase in Aβ species and in Aβ_42_/Aβ_40_ ratio [77], is a key factor exacerbating the effect of PSEN mutations on astrocyte Ca^2+^ homeostasis. This observation suggests a difference in acute and chronic effects of Aβ oligomers on astrocyte Ca^2+^ signals. In another mouse model, based on multiple mutations in APP (*APP^NL-F^*), the downregulation of astrocytic IP_3_R2 expression has been found associated with the reduction of Ca^2+^ signals in astrocytes from the CCx [61]. Notably, a downregulation in the expression of STIM1 and of IP_3_R2 has been reported also in post-mortem AD brains [61,62].

Overall, experimental results indicate that the accumulation of amyloid oligomers in the brain parenchyma, the establishment of a neuroinflammatory environment and the presence of pathogenic mutations together induce alterations in the intracellular Ca^2+^ signaling pathway of astrocytes and contribute to the development of AD phenotypes.

## 6. Conclusions

While this review aims to offer a comprehensive view of Ca^2+^ dysregulation in AD astrocytes, we acknowledge that the complexity of this multifactorial pathology cannot be captured by simplistic definitions. Undoubtedly, despite the discrepancies observed among mouse models and brain regions, we show that robust evidence of disrupted astrocyte Ca^2+^ homeostasis in AD pathology supports the presence of both hypo- and hyper-activity.

**Figure 2:**
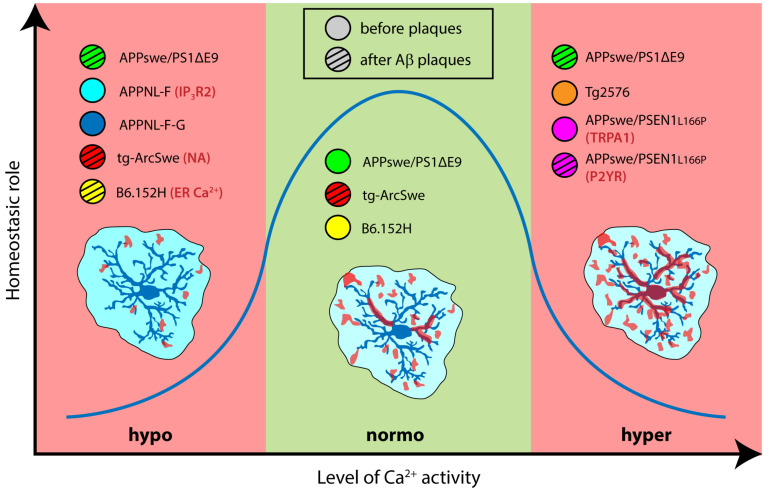
Schematic representation of the inverted U-shaped relationship (blue trace) showing that both reduced (hypo) and increased (hyper) Ca^2+^ activity negatively affect the homeostatic function of astrocytes. The position of the circles (color coded to identify the different AD mouse models) in the different sections indicates their Ca^2+^ phenotype, while their motif fill symbolizes the disease stage (uniform pattern = early stage, i.e., before plaque deposition; striped pattern = late stage, i.e., after plaque deposition). Where investigated, the mechanism underlying signal dysregulation is reported after the AD model designation.

The studies reported here comprise a collection of snapshots capturing astrocyte Ca^2+^ signals in different moments along the lifetime (young or adult mice) and AD progression (early or late pathology). Moreover, these views can focus on various details (brain regions) from diverse subjects (animal models characterized by distinct cellular expression of different AD mutations) and during discrete activity states (spontaneous or evoked signals). This fragmented information hampers the development of an integrative mechanistic model including all the variables and capable of predicting the functional consequences at synaptic and cognitive levels. However, these snapshots allow us to sketch a larger picture in which Ca^2+^ signalling is dynamically linked to the fundamental homeostatic role of astrocytes, following an inverted U-shape relationship where too little or too much signaling are equally detrimental for brain function (Figure 2).

Notably, astrocyte signal dysregulation including both hyper- or hypoexcitability has been reported in animal models of several other disorders affecting the nervous system, including Parkinson’s disease, Huntington’s disease, Down Syndrome, Amyotrophic Lateral Sclerosis and Major Depressive Disorder. These findings urge the scientific community to strengthen the investigation of the diverse molecular mechanisms underlying the disruption of Ca^2+^ homeostasis in astrocytes, with the aim of identifying therapeutic strategies capable of restoring their central modulatory role.

## Data Availability

No new data were created.

## Abbreviations

The following abbreviations are used in this manuscript:

α1-ARs	Adrenergic receptors
AD	Alzheimer’s disease
ApoE4	Apolipoprotein E
APP	Amyloid precursor protein
ATP	Adenosine triphosphate
Aβ	Amyloid beta
BDNF	Brain-derived neurotrophic factors
cAMP	Cyclic adenosine monophosphate
CCX	Cingulate cortex
CICR	Ca^2+^-induced Ca^2+^ release
CNO	Clozapine N-oxide
CRAC	Ca^2+^ release-activated Ca^2+^
DAG	Diacylglycerol
DREADDs	Designer Receptors Exclusively Activated by Designer Drugs
EOFAD	Early-onset familial AD
ER	Endoplasmic reticulum
FC	Functional connectivity
FLIM	Lifetime imaging microscopy
GABA	Gamma-aminobutyric acid
GLT-1	Glutamate transporter 1
GPCRs	G protein-coupled receptors
IP_3_	Inositol 1,4,5-trisphosphate
IP_3_Rs	IP_3_ receptors
LOAD	Late-onset AD
LTP	Long-term potentiation
MAPT	Microtubule-Associated Protein Tau
mGluRs	Metabotropic glutamate receptors
MLA	Methyllycaconitine
NA	Noradrenaline
NCX	Na^+^/Ca^2+^ exchanger
OGB-1	Oregon Green BAPTA-1
P2YRs	Purinergic receptors
PIP_2_	Phosphatidylinositol 4,5-bisphosphate
PLC	Phospholipase C
PPADS	Pyridoxalphosphate-6-azophenyl-2′,4′-disulfonic acid
PrP	Hamster prion protein
PS1	Presenilin 1
PS2	Presenilin 2
ROA	Region of activity
ROI	Region of interest
RyRs	Ryanodine receptors
SERCA	Sarco-Endoplasmic Reticulum Calcium ATPase
SOCE	Store-operated Ca^2+^ entry
SR101	Sulforhodamine 101
SSCx	Somatosensory cortex
STIM	Stromal interaction molecule
SWA	Slow-wave activity
WT	Wild type
